# Differences in SMA-like polymer architecture dictate the conformational changes exhibited by the membrane protein rhodopsin encapsulated in lipid nano-particles†

**DOI:** 10.1039/d1nr02419a

**Published:** 2021-08-02

**Authors:** Rachael L. Grime, Richard T. Logan, Stephanie A. Nestorow, Pooja Sridhar, Patricia C. Edwards, Christopher G. Tate, Bert Klumperman, Tim R. Dafforn, David R. Poyner, Philip J. Reeves, Mark Wheatley

**Affiliations:** School of Biosciences, University of Birmingham Birmingham B15 2TT UK; Centre of Membrane Proteins and Receptors (COMPARE), University of Birmingham and University of Nottingham Midlands UK; MRC Laboratory of Molecular Biology Francis Crick Avenue Cambridge CB2 0QH UK; Department of Chemistry and Polymer Science, Division of Polymer Science, Stellenbosch University Private Bag X1 Matieland 7602 South Africa; Life and Health Sciences, Aston University Birmingham B4 7ET UK; School of Life Sciences, University of Essex Wivenhoe Park Essex CO4 3SQ UK preeves@essex.ac.uk; Centre for Sport, Exercise and Life Sciences, Institute for Health & Wellbeing, Alison Gingell Building, Coventry University Coventry CV1 2DS UK mark.wheatley@coventry.ac.uk

## Abstract

Membrane proteins are of fundamental importance to cellular processes and nano-encapsulation strategies that preserve their native lipid bilayer environment are particularly attractive for studying and exploiting these proteins. Poly(styrene-*co*-maleic acid) (SMA) and related polymers poly(styrene-*co*-(*N*-(3-*N*′,*N*′-dimethylaminopropyl)maleimide)) (SMI) and poly(diisobutylene-*alt*-maleic acid) (DIBMA) have revolutionised the study of membrane proteins by spontaneously solubilising membrane proteins direct from cell membranes within nanoscale discs of native bilayer called SMA lipid particles (SMALPs), SMILPs and DIBMALPs respectively. This systematic study shows for the first time, that conformational changes of the encapsulated protein are dictated by the solubilising polymer. The photoactivation pathway of rhodopsin (Rho), a G-protein-coupled receptor (GPCR), comprises structurally-defined intermediates with characteristic absorbance spectra that revealed conformational restrictions with styrene-containing SMA and SMI, so that photoactivation proceeded only as far as metarhodopsin-I, absorbing at 478 nm, in a SMALP or SMILP. In contrast, full attainment of metarhodopsin-II, absorbing at 382 nm, was observed in a DIBMALP. Consequently, different intermediate states of Rho could be generated readily by simply employing different SMA-like polymers. Dynamic light-scattering and analytical ultracentrifugation revealed differences in size and thermostability between SMALP, SMILP and DIBMALP. Moreover, encapsulated Rho exhibited different stability in a SMALP, SMILP or DIBMALP. Overall, we establish that SMA, SMI and DIBMA constitute a ‘toolkit’ of solubilising polymers, so that selection of the appropriate solubilising polymer provides a spectrum of useful attributes for studying membrane proteins.

## Abstract 2

Using the GPCR rhodopsin as an exemplar, SMA SMI and DIBMA constitute a ‘tool-kit’ of structurally-related solubilising polymers, with each providing different advantages for studying membrane proteins encapsulated in lipid particles.

## Graphical abstract

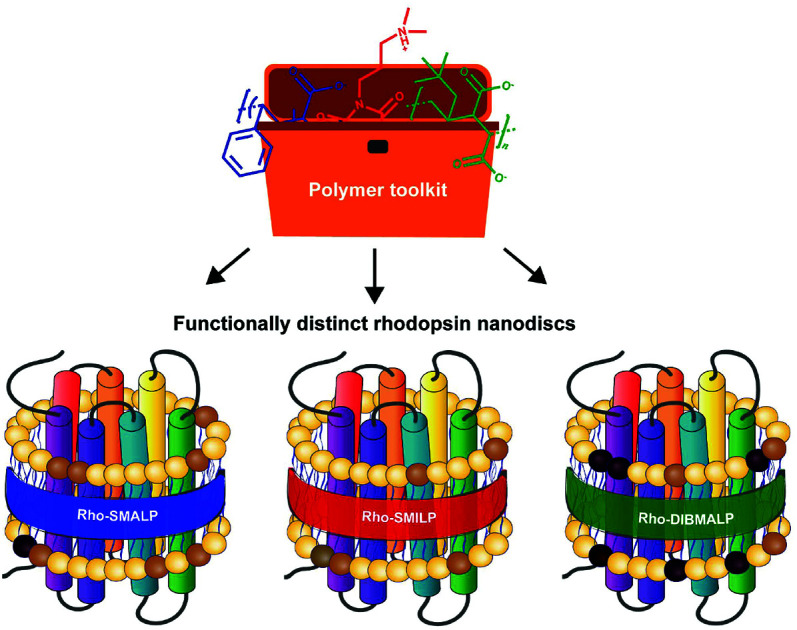

## Introduction

Membrane proteins have evolved to function in the specialised environment of a hydrated lipid bilayer membrane. This subjects the embedded protein to lateral pressure and places it in close association with lipids. Extracting membrane proteins for study has until recently, universally required detergents. However, the detergent micelle is often a poor mimic of the native bilayer, lateral pressure is lost and annular lipids in close proximity to the protein are stripped away, resulting in conformational change and protein instability.^1,2^ In recent years there has been a rapid increase in the use of detergent-free methods for solubilising membrane proteins. Poly(styrene-*co*-maleic acid) (SMA) spontaneously incorporates into membranes and generates a nanoscale section of native membrane as a disc (∼10 nm in diameter), referred to as a styrene maleic acid lipid particle (SMALP), stabilised by a belt of polymer and encapsulating membrane protein.^3–8^ SMALPs are proving to be a versatile platform for studying membrane proteins, facilitating biophysical investigation^9–11^ plus structural and functional studies,^6,12–15^ and with potential for high-throughput screening in drug discovery following immobilisation of SMALP-encapsulated therapeutic target proteins, such as receptors, on surface plasmon resonance (SPR) chips.^8^ This expanding utility of SMALPs has been the driver for developing ‘second generation’ SMA-like polymers, including poly(styrene-*co*-(*N*-(3-*N*′,*N*′-dimethylaminopropyl)maleimide)) (SMI)^16^ and poly(diisobutylene-*alt*-maleic acid) (DIBMA).^17^ In common with SMA, both SMI and DIBMA directly solubilise cell membranes to generate nanoscale lipid particles analogous to SMALPs referred to as SMILPs and DIBMALPs respectively.^16,18^ Although progress has been made in characterising the differences in architecture and biophysical properties between SMALPs, SMILPs and DIBMALPs,^5,16–19^ a fundamental question that has not been addressed to-date, is whether the structure of the polymer stabilising the lipid particle affects the conformational changes of the membrane protein encapsulated within it.

In this study, we report the first systematic investigation into the effect of the polymer structure on the range of conformational changes exhibited by a dynamic protein (rhodopsin, the dim light receptor in the eye activated by light) when encapsulated in a SMALP, SMILP or DIBMALP. Rhodopsin is a G-protein-coupled receptor (GPCR). GPCRs are central to cell signalling throughout the evolutionary tree from humans to viruses (but not bacteria) and form the largest class of ‘chemical switches’ in biology. They transduce signals from chemical messengers acting on a cell, such as hormones and neurotransmitters, into biochemical changes within the cell *via* activation of intracellular signalling cascades.^20^ As a result, they regulate almost every physiological process. Consequently, GPCRs form the largest family of membrane proteins in the human genome (with >800 receptors) and are very important to the pharmaceutical industry as they are the therapeutic target of 30–40% of clinically-prescribed drugs.^21,22^ GPCRs share a common protein architecture of a bundle of seven transmembrane helices (TMs) and adopt a wide spectrum of conformational states in executing their cell signalling role.^23^ Rhodopsin is an archetypical GPCR for which the intermediate structures between the inactive and active states have been defined in great detail.^24^ This makes rhodopsin an excellent ‘probe’ to investigate the effect of the chemical composition of the stabilising polymer belt of structurally-related ‘SMALP-like’ lipid particles on the conformational changes of the encapsulated membrane protein.

Our study establishes for the first time that the choice of polymer used to solubilise a membrane protein dictates the changes in conformation observed for the encapsulated protein. Moreover, these different polymers effectively provide a ‘toolkit’ that can be exploited by researchers to study different conformational states and provide a range of stabilities that expand the range of down-stream applications and allow the investigator to tailor the properties of SMALP-like lipid particle to their research needs.

## Experimental

### Preparation of rod outer segment (ROS) from bovine retina

Rhodopsin ROS was prepared from bovine retina by sucrose density gradient centrifugation of ROS disc membranes, as described previously.^25^ Bovine retinas were purchased from W.L. Lawson Company Nebraska and stored frozen at −80 °C. Retinas were defrosted and resuspended into buffer A (70 mM K_3_PO_4_, 1 mM MgCl_2_, 5 mM BME, 0.1 mM PMSF, 30% sucrose, pH 6.8) for ROS extraction. Homogenates were centrifuged at 4500*g* for 6 min at 4 °C. Pellets were re-extracted twice more into buffer A. The pellet was then resuspended into buffer B (70 mM K_3_PO_4_, 1 mM MgCl_2_, 5 mM BME, 0.1 mM PMSF, pH 6.8) and centrifuged at 50 000*g* for 20 min at 4 °C. The supernatant was discarded and ROS pellets were pooled and resuspended into buffer C (70 mM K_3_PO_4_, 1 mM MgCl_2_, 5 mM BME, 0.1 mM PMSF, 15% sucrose, pH 6.8). The suspension was divided equally into centrifuge tubes, and under-laid with buffer D (70 mM K_3_PO_4_, 1 mM MgCl_2_, 5 mM BME, 0.1 mM PMSF, 0.64 M sucrose, pH 6.8), using a syringe and long needle. The sample was centrifuged at 50 000*g* for 20 min at 4 °C. The supernatant was removed and pellets were resuspended and homogenised into buffer D (70 mM K_3_PO_4_, 1 mM MgCl_2_, 5 mM BME, 0.1 mM PMSF, 0.64 M sucrose, pH 6.8). Sucrose step gradients were then prepared in Ultra-clear tubes (Beckman PA UZ-PA-38-5-1). Sucrose density gradients were 0.78 M, 1 M and 1.2 M in 70 mM K_3_PO_4_, 1 mM MgCl_2_, 5 mM BME, 0.1 mM PMSF, pH 6.8. ROS was over-laid onto the gradient. Samples were centrifuged (with no brake) at 100 000*g* for 45 min at 4 °C. The ROS fraction was recovered by puncturing the thin wall tube by the 0.78 M/1 M interface and aspirating with an #18-gauge needle. The recovered ROS was diluted in buffer B and centrifuged at 125 000*g* for 20 min at 4 °C. The supernatant was discarded and resuspended and homogenised into buffer B, then centrifuged again at 125 000*g* for 20 min at 4 °C. This wash step was performed twice. ROS pellet was then resuspended and homogenized into buffer E (50 mM Tris-HCl, 5 M urea, 5 mM EDTA, pH 7.4). The homogenate was incubated for 1 h, at 4 °C on an end-over-end rotator. Following dilution into buffer F (50 mM Tris-HCl, pH 7.4) ROS membranes were centrifuged at 125 000*g* for 45 min at 4 °C. The supernatant was removed and a series of three repeated washes removed urea traces (resuspension in buffer F, followed by centrifugation at 125 000*g* for 30 min at 4 °C). The final ROS pellet was resuspended and homogenised into buffer G (20 mM Tris-HCl, 2 mM EDTA, 10% sucrose, 1 mM BME, 0.1 mM PMSF, pH 7.2). Aliquots were snap-frozen and stored at −80 °C wrapped in foil.

### Preparation of amphipathic copolymers

SMA was prepared from poly(styrene-*co*-maleic anhydride) (SMAnh) (purchased from Cray Valley, UK) exactly as described previously.^7^ For SMA preparation, a 10% (w/v) solution of SMAnh was dissolved in 1 M NaOH and heated under reflux at 125 °C for 2–4 h, until the solution clarified. Solubilised SMA was then precipitated by drop-wise addition of concentrated HCl and washed three times in ultrapure water. The SMA was then re-dissolved in 0.6 M NaOH and wash steps repeated. Finally, SMA was re-dissolved in 0.6 M NaOH, the pH adjusted to pH 8.0 and lyophilised. SMI was prepared from SMA2000I resin (purchased from Cray Valley, UK), exactly as described previously.^16^ Briefly, for SMI preparation, concentrated HCl was added dropwise to 10% (w/v) SMA2000I in ultra-pure water to yield a 1 M solution. The solution was heated under reflux at 125 °C for 2–4 h, until clarification. Solubilised SMI was then precipitated by addition of 5 M NaOH to pH 9.0 and washed three times in ultrapure water. The precipitated SMI was then re-dissolved in a minimal volume of 0.6 M HCl, and pH adjusted to pH 6.0 before lyophilisation. DIBMA was prepared from Sokalan CP9. Briefly, 10 mL of Sokalan was pipetted into 40 cm of dialysis tubing (1000 MWCO) and dialysed for 24 h in dH_2_0 water, with a water change at 12 h. A second dialysis stage was then performed, for a further 24 h (with water change at 12 h), with ∼200 mL solution being placed into 80 cm fresh dialysis tubing (1000 MWCO). After 24 h, the solution was pH adjusted to pH 8.0, then frozen at −80 °C for 48 h and subsequently lyophilised.

### Solubilisation of ROS with SMA, SMI and DIBMA

ROS membranes preparations were thawed on ice and approximately 100 μg protein was added to relevant extraction buffers. The DDM extraction buffer consisted of 1% (w/v) DDM in 1× PBS, pH 7.8. The SMA extraction buffer consisted of 2.5% (w/v) SMA in 1× PBS, pH 7.8. The SMI extraction buffer consisted of 2.5% (w/v) SMI in 1× PBS, pH 6.8. The DIBMA extraction buffer consisted of 5% (w/v) DIBMA in 1× PBS, pH 7.8. Samples were left on an end-over-end rotator for 1 h (room temperature) before centrifugation at 100 000*g*, 1 h, 4 °C. Supernatant containing solubilised rhodopsin was collected and stored at 4 °C. Insoluble pellets were resuspended in an equal volume of 1× PBS, and stored at 4 °C. Rhodopsin solubilisation efficiency was determined using UV-visible absorption spectroscopy.

### UV-visible absorption spectroscopy of rhodopsin and photobleaching

UV-visible (UV-VIS) absorption spectroscopy was performed using a Perkin-Elmer λ35 UV-VIS spectrophotometer equipped with water-jacketed cell holder. All scans were performed at 20 °C, unless otherwise stated, and temperatures in the quartz cuvettes were confirmed using a thermocouple probe (Omega HH800A). Temperature was regulated with a water bath (Grant LTCI GD120). A scan speed of 480 nm min^−1^ was set, as well as a data interval of 2 nm bandwidth, with a response time of 1 s. All spectra obtained were subject to normalisation to correct for baseline-drift, whereby absorbance at 650 nm (*A*_650_) was corrected to exactly zero. For photobleaching, all samples were illuminated directly in the spectrophotometer quartz cuvette using a fibre-optic light guide (SCHOTT KL1500 Compact) fitted with a >495 nm long-pass filter. Full dark-state spectra were obtained before manually photobleaching the samples for 30 s intervals, for up to 150 s. To determine solubilisation efficiency, quantitative yield was calculated based on the baseline corrected absorbance at 500 nm (*A*_500_). The molar extinction coefficient value used for rhodopsin at 500 nm was 40 600 M^−1^ cm^−1^.^26^ Rhodopsin yields were then determined using The Beer–Lambert Law (eqn (1)); where *ε* is molar extinction coefficient (M^−1^ cm^−1^); *c* is concentration (*M*); and *l* is the path length (cm). Molar concentration (*c*) was then converted to mass using eqn (2) (relative formula mass of bovine rhodopsin = 39 010 dA).1
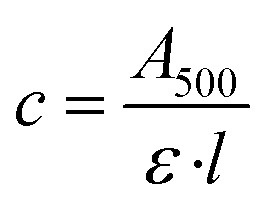

2 Mass = *c* × *M*_r_

### Thermal stability of encapsulated rhodopsin

Thermal stability of rhodopsin encapsulated in a lipid particle (Rho-LP; either SMALP, SMILP or DIBMALP) was determined by monitoring *A*_500_ over time at the stated temperature. *A*_500_ was monitored *via* UV-VIS absorption spectroscopy as described above. To determine the stability of the Rho-LP at 37 °C, the temperature regulating water bath was set to provide an in-cuvette temperature of 37 °C. Cuvettes were pre-heated prior to addition of Rho-LP sample and then allowed to equilibrate in the spectrophotometer for 90 s, before the absorbance spectrum was recorded between 650 nm and 250 nm. Spectra were recorded every 30 min for a total of 960 min. Data were then normalised to allow for plotting of relative *A*_500_ (%). *A*_500_ at 0 min was corrected to 100%, and 0% was set to the *A*_500_ value of the buffer blank. When the stability of the Rho-LP was determined over a range of temperatures, the samples were incubated at the stated temperatures for 30 min before the UV-VIS absorption spectrum was recorded at 20 °C, as described above.

### Analytical ultracentrifugation (AUC)

Rho-LP samples were prepared for AUC in 137 mM NaCl, 2.7 mM KCl and 10 mM phosphate buffer, pH 7.4. Experiments were performed using a Beckman Coulter ProteomeLab XL-I Analytical Ultracentrifuge with a Ti50 rotor at 20 °C, 40 000 rpm (129 000*g*). Absorbance at 280 nm was monitored. Data were analysed using SEDFIT applying the continuous c(s) distribution model.^27^ Frictional ratio was permitted to float.

### Dynamic light scattering (DLS)

Dynamic light scattering (DLS) experiments were preformed using a DynaPro Plate Reader III and DYNAMICS software (Wyatt Technology, Haverhill, UK), using the laser wavelength of 825.4 nm with a detector angle of 150°. Each sample (40 μL) was loaded into a 384-well glass bottom SensoPlate™ (Greiner Bio-One, Germany) in triplicate. Each measurement consisted of 5 scans of 5 s. Scans were carried out initially at 25 °C, with discrete 5 °C temperature increases, up to 60 °C. Each stepped-increase in temperature was maintained for 30 min before DLS data were collected to establish equilibrium. The attenuator position and laser power automatically optimised for size (nm) determination.

## Results and discussion

### The solubilising polymer dictates the range of conformational changes in the encapsulated protein

**Fig. 1:**
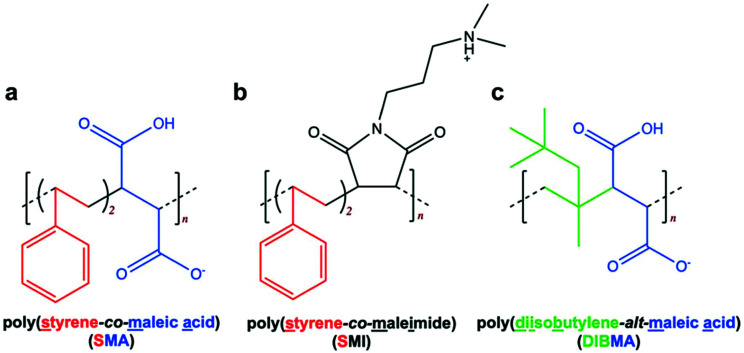
Structurally-related polymers used to solubilise membrane proteins. (a) SMA, (b) SMI and (c) DIBMA.

SMA (Fig. 1a) revolutionised the study of membrane proteins, by spontaneously solubilising membrane proteins within nanoscale discs of native bilayer called SMALPs. ‘Second generation’ SMA-like polymers SMI and DIBMA retain the ability of SMA to spontaneously solubilise membrane proteins but exhibit different physico-chemical characteristics to SMA. SMI possesses the styrene aromatic ring of SMA but is positively-charged (Fig. 1b) and retains functionality at acidic pH, in contrast to the negative charge and alkaline working range of SMA.^16^ DIBMA has the styrene aromatic ring of SMA replaced by aliphatic diisobutylene but retains the maleic acid component of SMA (Fig. 1c).^17^

In order to investigate the effect of the chemical composition of the polymer belt on the dynamic properties of the encapsulated protein, we characterised photoactivation of rhodopsin encapsulated in a SMALP, SMILP or DIBMALP. Activation of rhodopsin has been defined in great detail and occurs *via* a series of specific intermediates.^24^ The photoreactive chromophore of rhodopsin is 11-*cis* retinal covalently bound to Lys296 by a protonated Schiff base. Absorption of a photon of light induces rapid isomerisation of 11-*cis* retinal to the all-*trans* configuration, a potent agonist. This drives the conversion of dark-adapted inactive rhodopsin through a series of structurally-defined intermediates, each with its characteristic absorption maximum (*λ*_max_), to the fully-activated conformation metarhodopsin II (Meta II) which absorbs at 382 nm (Fig. S1†).^24^ In addition to crystal structures of the dark inactive rhodopsin and fully active Meta II, there are crystal structures of the intermediate states bathorhodopsin, lumirhodopsin and Meta I^28–31^ making rhodopsin the most structurally-defined GPCR. These structures reveal that rhodopsin activation is not a gradual build-up of conformational change, as there is relatively little change in the overall structure of light-activated rhodopsin as it transitions between the photointermediates from the dark ground-state as far as Meta I. In contrast however, conversion of Meta I to Meta II is accompanied by a large conformational change at the cytoplasmic end of transmembrane helix-6 (TM6), which moves out from the helical bundle, opening a binding crevice to allow docking of the G-protein, transducin (G_t_).^32,33^ This large outward movement of the cytoplasmic end of TM6 (by 7–14 Å) is a characteristic feature of full activation of family A GPCRs in general.^23,34^ This detailed knowledge of the structural and spectroscopic features of the conformational states of rhodopsin during its photoactivation means that we can track the sequential conformational changes from the inactive dark state to the fully active Meta II conformation. Rhodopsin is therefore an excellent ‘probe’ to investigate the effect of the chemical composition of the stabilising polymer belt of a ‘SMALP-like’ lipid particle on the conformational changes of the encapsulated membrane protein. Furthermore, using bovine retina rhodopsin as the encapsulated membrane protein allowed us to use the wild-type protein resident in rod cell disc membranes and avoided the use of an engineered recombinant construct with introduced reporter groups, or structural changes, that might affect conformational changes.

**Fig. 2:**
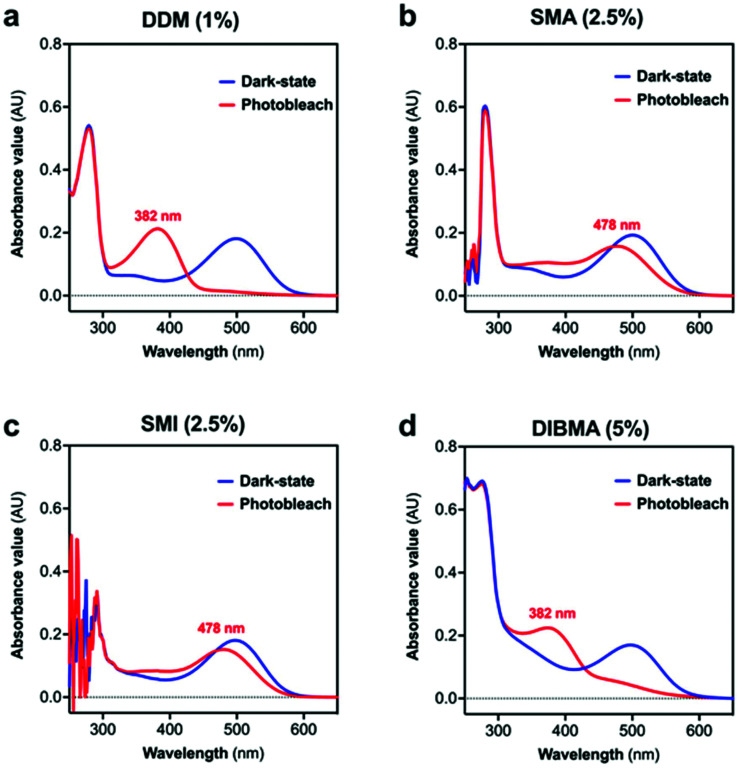
Spectra of dark-state and photoactivated rhodopsin in (a) DDM, (b) SMALP, (c) SMILP and (d) DIBMALP.

Dark-adapted rhodopsin possesses a characteristic absorption peak (*λ*_max_) at 500 nm,^24^ and this peak was clearly evident when rhodopsin was solubilised by either the widely-used detergent *n*-dodecyl-β-d-maltopyranoside (DDM) or by encapsulation in one of the polymer-stabilised lipid particles (SMALP, SMILP or DIBMALP) (Fig. 2a–d). The efficiency of solubilisation of rhodopsin was similar for DDM (1%), SMA (2.5%), SMI (2.5%) and DIBMA (5%), where the number in the brackets is the concentration used in each case. The extraction efficiency values relative to DDM were 99 ± 5.5%, 97 ± 2.6% and 98 ± 4.8% (mean ± s.e.m., *n* = 3) for SMA, SMI and DIBMA respectively. Optimisation of rhodopsin solubilisation by DIBMA (Table S1†) revealed that a slightly higher concentration of DIBMA (5%) was required to generate the same extraction efficiency as SMA (2.5%) and SMI (2.5%). Photoactivation (30 s, *hν* > 495 nm) of the DDM-solubilised rhodopsin generated the characteristic changes in the rhodopsin spectrum, with loss of the peak at 500 nm corresponding to ground-state rhodopsin and appearance of a new absorption peak (*λ*_max_) at 382 nm (Fig. 2a), consistent with the formation of the fully-active rhodopsin conformation Meta II (Fig. S1†).^24^ Likewise, photoactivation of rhodopsin encapsulated in a SMALP or SMILP caused a loss of the peak at 500 nm but in contrast to DDM, generated a new absorption peak with *λ*_max_ = 478 nm rather than 382 nm (Fig. 2b and c). This revealed that photoconversion of rhodopsin in a SMALP or SMILP proceeded only as far as Meta I (*λ*_max_ = 478 nm; Fig. S1†) but did not progress further to the active Meta II conformation (*λ*_max_ = 382 nm), even after an extended photobleaching period of 150 s (Fig. 2b and c). Both SMA and SMI contain styrene rings which give absorption in the UV region (<300 nm) of the spectrum.^16,17^ In marked contrast to SMALP or SMILP, photoactivation of rhodopsin encapsulated in a DIBMALP progressed beyond Meta I to the active Meta II conformation (*λ*_max_ = 382 nm; Fig. 2d). The slight absorption observed >430 nm may indicate the presence of low levels of residual photointermediates. The slightly higher absorbance <400 nm in the dark-state Rho-DIBMALP spectrum compared to Rho-SMALP and Rho-SMILP, is probably due to greater light-scattering by the larger DIBMALP.

The active conformation of rhodopsin can be stabilised by occupation of the G-protein binding crevice on the cytoplasmic side of the receptor. This can be achieved by incubation with a small 11-mer peptide corresponding to the C-terminus sequence of the α5 helix of the α-subunit of transducin, referred to as G(t)-peptide^35,36^ or by formation of a rhodopsin:mini-G_o_ complex.^34^ Mini-G proteins comprise the guanosine triphosphatase (GTPase) domain of the α-subunit of heterotrimeric G-proteins and are fully capable of stabilising GPCRs in their active state.^37^ Although full activation of rhodopsin to Meta II was not observed in SMALP or SMILP, it was feasible that rhodopsin in a SMALP or SMILP could transition inefficiently to Meta II but at levels too low to detect. To address this, photoactivation was performed in the presence of Gt peptide or mini-G_o_ to trap any Meta II formed. However, neither G(t)-peptide (500 μM) nor mini-G_o_ (present in 500-fold molar-excess) increased Meta II formation (Fig. S2†). Therefore, it is highly unlikely that rhodopsin encapsulated in a SMALP or SMILP can transition to the Meta II conformation. It is noteworthy that the two polymers that prevented Meta II formation (SMA and SMI) both possess a styrene aromatic ring but when this is replaced by aliphatic diisobutylene in DIBMA (Fig. 1) rhodopsin can transition to the active Meta II conformation. It is known that the phenyl rings of the styrene moieties in the polymer belt intercalate between the lipid acyl chains perpendicular to the plane of the lipids,^5^ which can affect the lipid packing order and the transition temperature for gel to liquid phase transitions.^17^ This could have important functional ramifications as membrane fluidity could impact on the conformational flexibility of dynamic membrane proteins like GPCRs, including rhodopsin. Interestingly, rhodopsin solubilised by the detergent digitonin also gets trapped at Meta I upon activation possibly due to the digitonin micelle forming a more rigid environment.^38^ In contrast, solubilisation by DIBMA has been shown to cause very little perturbation to the packing and phase transition temperature of the lipids.^17^ Furthermore, electron paramagnetic resonance spectroscopy and course-grained molecular dynamics simulations revealed that the dynamics of lipids in a DIBMALP are less constrained than in a SMALP and the affinity of spin-labelled lipids for the polymer belt was more pronounced in a SMALP.^39^ There are also differences in lipid transfer, which is slower among DIBMALPs than among SMALPs.^40^ The current study shows that although light-induced formation of Meta I occurs within a SMALP or SMILP environment, there is some degree of conformational restriction endowed by the nature of the copolymer belt that prevents the full activation of rhodopsin to Meta II observed within a DIBMALP. The suggestion that the copolymer component of a SMALP or SMILP can restrict the range of conformational transitions adopted by the encapsulated protein, either directly or *via* changes in lipid packing and dynamics, is supported by recent studies on the human adenosine A_2A_ receptor (A_2A_R) and the photoreceptor/transducer complex (*Np*SRII2/*Np*HtrII2) from *Natronomonas pharaonisfrom*.^11,41^ Stimulation of A_2A_R in a SMALP by the agonist NECA generated only small conformational changes^11^ and the conformational freedom of *Np*SRII2/*Np*HtrII2 was also restricted in a SMALP.^41^ Interestingly, *Np*SRII2 could be fully activated when encapsulated in a DIBMALP.^42^

### Characterisation of Rho-SMALP, Rho-SMILP and Rho-DIBMALP

**Fig. 3:**
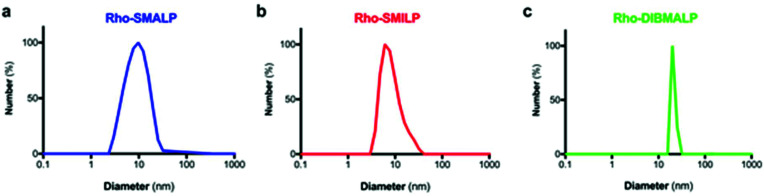
Size of the Rho-LPs determined by DLS. A representative experiment is shown for (a) Rho-SMALP, (b) Rho-SMILP and (c) Rho-DIBMALP.

**Fig. 4:**
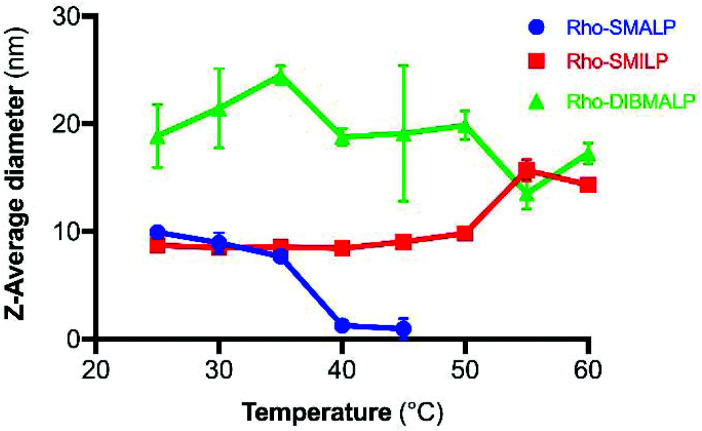
Temperature-dependent changes in Rho-LP diameter determined by DLS, for Rho-SMALP (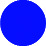), Rho-SMILP (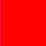) and Rho-DIBMALP (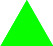). Data are mean ± s.e.m. (*n* = 3).

The characteristics of the rhodopsin lipid particles (Rho-LPs) were investigated further. Rhodopsin encapsulated in a SMALP, SMILP and DIBMALP (Rho-SMALP, Rho-SMILP, Rho-DIBMALP respectively) was analysed by dynamic light scattering (DLS) to determine the hydrodynamic diameter of the particles (Fig. 3a–c). The size of Rho-SMALP and Rho-SMILP were similar with *z*-average diameters of 9.9 ± 0.2 nm, and 8.7 ± 0.3 nm (mean ± s.e.m., *n* = 3) respectively, consistent with previously published values of ∼10 nm diameter.^3,5,16,43^ The Rho-DIBMALP was larger, with a *z*-average diameter of 18.9 ± 2.9 nm (mean ± s.e.m., *n* = 3) which is also in agreement with published values.^17,44^ It is possible that the larger diameter of the DIBMALP compared to the SMALP and SMILP contributes to its ability to support transition to Meta II, as computational simulations of lipid dynamics in empty ‘lipid only’ discs indicated a stiffening of the lipids due to a confinement effect of the disc but this stiffening was lower when the disc diameter was increased from 9.8 nm to 18.4 nm.^45^ The thermostability of the Rho-LPs was determined using DLS in combination with increasing the temperature by 5 °C intervals from 25 °C up to a maximum of 60 °C. Clear differences were observed between the different lipid particles (Fig. 4). Rho-SMALP was the least stable, with a loss of DLS signal >40 °C suggesting breakdown of the Rho-SMALP at these higher temperatures. In contrast, both Rho-SMILP and Rho-DIBMALP were stable over the temperature range tested. An apparent increase in the *z*-average diameter of Rho-SMILP from 8.7 ± 0.3 nm to 15.0 ± 0.9 nm (mean ± s.e.m., *n* = 3) was noted >50 °C (Fig. 4). It can be speculated that this represents interaction between individual Rho-SMILPs at the higher temperatures.

In the current study, encapsulation in a DIBMALP provided rhodopsin with a greater degree of thermostability than in a SMALP (Fig. 6a and b). However, it appears that there are protein-specific differences with respect to the comparative stability of proteins encapsulated in a SMALP *versus* a DIBMALP. For example, the thermostability of the human serotonin transporter (hSERT) is comparable in a SMALP or a DIBMALP but the A_2A_R in a DIBMALP was reported to be less stable to long-term storage (6 days) at 4 °C than in a SMALP.^44,46^

**Fig. 5:**
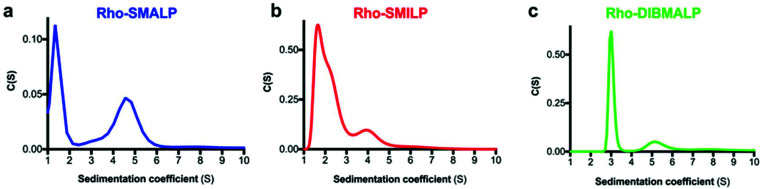
Sedimentation velocity AUC analysis of Rho-LPs. A representative AUC experiment is shown for (a) Rho-SMALP, (b) Rho-SMILP and (c) Rho-DIBMALP.

**Fig. 6:**
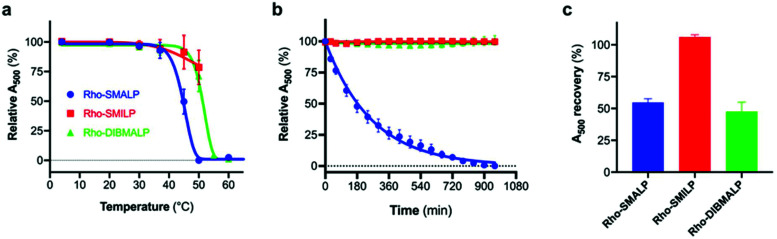
Stability of the encapsulated Rho in Rho-LPs. The decay of Rho encapsulated in SMALP (blue), SMILP (red) or DIBMALP (green) was monitored by decrease in absorbance at 500 nm. (a) Thermostability was assessed after incubating the Rho-LPs for 30 min at the temperatures indicated. (b) Thermostability at 37 °C. (c) Recovery following lyophilisation and rehydration. Data are mean ± s.e.m. (*n* = 3) in each case.

Rho-LP were characterised further using sedimentation velocity analytical ultracentrifugation (AUC; Fig. 5). The data were analysed using the continuous c(s) analysis method to determine sedimentation coefficients and molar masses, using the SEDFIT software and the method of Schuck.^27^ The large peak in each trace, with sedimentation coefficients of ∼1.5S (Rho-SMALP; Fig. 5a), ∼2S (Rho-SMILP; Fig. 5b) and 3S (Rho-DIBMALP; Fig. 5c) respectively, is likely to be free polymer and polymer aggregates. The second peak in each trace is compatible with the known sizes of the Rho-LPs. The sedimentation coefficient of 4.6S for Rho-SMALP is consistent with a molar mass of ∼89 kDa. It has been determined previously that the SMA polymer and lipid of a SMALP contribute ∼35 kDa of a ∼70 kDa SMALP encapsulating the protein ZipA.^7^ This would imply that the Rho component of Rho-SMALP is ∼54 kDa. Given that the mass of Rho, including the post-translational modifications (two glycosylation sites plus two palmitoylation sites) is ∼43 kDa,^47^ this would indicate that the encapsulated Rho was a monomer and not a dimer (∼86 kDa) or higher oligomer. This is relevant as although it is known that a single light-activated Rho is sufficient to activate its G-protein transducin,^48^ in native rod outer segment membranes Rho can organise into rows of dimers.^49,50^ The fact that monomeric Rho and not Rho dimers were encapsulated in the SMALP might be a ramification of the precise molecular mechanisms by which SMA monomers are incorporated into the membrane and polymerise to excise the SMALPs. What is clear, is that encapsulation of Rho monomers was not due to there being insufficient space within a SMALP to accommodate the 14-transmembrane helices (TMs) of a rhodopsin dimer, as Alternative Complex III in a functional super-complex with cytochrome oxidase was isolated encapsulated within a SMALP despite having a total of 48-TMs.^12^ The DLS experiments presented earlier in this study highlighted that Rho-SMALP and Rho-SMILP were of similar size. AUC revealed that Rho-SMILP had a sedimentation coefficient 4.1S consistent with a molar mass of ∼87 kDa. Lipid-only ‘empty’ SMILP containing only 1,2-dimyristoyl-*sn-glycero*-3-phosphocholine (DMPC-SMILP) without encapsulated protein gave a single major peak (2.35 S, Fig. S3†) consistent with a molar mass of 56 kDa, with a minor component at ∼5S. This is 31 kDa less than the Rho-SMILP, suggesting encapsulation of a single Rho (∼43 kDa), like Rho-SMALP, and not a dimer (∼86 kDa). DLS revealed that Rho- DIBMALP is larger than either Rho-SMALP or Rho-SMILP and this was apparent in the AUC as Rho-DIBMALP had a sedimentation coefficient of 5.2 S and a predicted molar mass of 117 kDa. Lipid-only ‘empty’ DIBMALP (DMPC-DIBMALP) gave a single major AUC peak (3.35 S, Fig. S3†) consistent with a molar mass of 65 kDa, with a minor component at ∼5S. This is 52 kDa less than the Rho-DIBMALP, again suggesting encapsulation of a Rho monomer (∼43 kDa) and not a dimer (∼86 kDa). In addition to investigating the effect of temperature on the lipid particles (Fig. 4), the thermostability of the encapsulated protein within each Rho-LP was also compared. Each Rho-LP was incubated at a range of temperatures between 4 °C–60 °C, using 5 °C increments, and the thermal stability of Rho was determined by the decrease in absorbance at 500 nm which represents the release of the 11-*cis*-retinal chromophore (Fig. 6a). The Rho within a Rho-SMALP was the least thermostable with a *T*_50_ = 45.7 ± 0.5 °C (mean ± s.e.m., *n* = 3). Encapsulation of Rho in DIBMALP conferred an increase in Rho thermostability of 6 °C (*T*_50_ = 51.4 ± 0.8 °C mean ± s.e.m., *n* = 3) compared to Rho-SMALP (Fig. 6a). The lower thermostability of Rho in a SMALP compared to a DIBMALP may reflect the lower thermostability of the SMALP particle compared to the DIBMALP particle (Fig. 4). Rho was also more thermostable in a SMILP than a SMALP (Fig. 6a), although *T*_50_ = >50 °C, a precise *T*_50_ value could not be determined for Rho-SMILP as above 55 °C a slight precipitate formed which prevented data collection at higher temperatures. The rate of thermal decay of Rho was determined at 37 °C for each Rho-LP using a pre-heated spectrophotometer and monitoring absorbance at 500 nm for 16 h (Fig. 6b). Consistent with data presented above, the Rho encapsulated in a SMALP was the least thermostable at 37 °C (Rho-SMALP; *t*_1/2_ = 183 ± 5 min; mean ± s.e.m. (*n* = 3)). In contrast, Rho within a SMILP or a DIBMALP showed no thermal decay over the 16 h period. It is noteworthy that the rate of Rho decay in the Rho-SMALP at 37 °C was very similar to the loss of ligand binding capability of another GPCR, the human adenosine A_2A_ receptor (A_2A_R) encapsulated in a SMALP incubated at 37 °C (A_2A_R-SMALP; *t*_1/2_ = 148 ± 13 min; mean ± s.e.m. (*n* = 3)).^6^ As an extreme test of stability, the effect of lyophilisation on Rho-LPs was quantified using absorbance at 500 nm to assess Rho decay following lyophilisation and re-hydration with buffer (Fig. 6c). Despite such harsh treatment, ∼50% of the Rho encapsulated in a SMALP or DIBMALP was recovered with the chromophore intact (54 ± 3% and 48 ± 7% respectively, mean ± s.e.m. (*n* = 3)). However, Rho encapsulated in SMILP was even more stable and was unaffected by lyophilisation (recovery 106 ± 2%, mean ± s.e.m., *n* = 3; Fig. 6c). The availability of such a stable storage form of a membrane protein as a lyophilised SMILP could be a useful resource not only for storage but as a reagent for downstream applications.

Defining the characteristics of Rho, a native GPCR, encapsulated by three different but structurally-related polymers (Rho-SMALP, Rho-SMILP and Rho-DIBMALP) enabled direct comparisons to be made. SMALP and SMILP allowed conformational change in light-activated Rho as far as Meta I but the effect of the styrene ring in these polymers, or constraints due to their smaller size, prevented complete photoconversion of Rho to the fully-active Meta II observed in Rho-DIBMALP. Consequently, SMALP and SMILP, both of which prevented full activation of Rho, might facilitate structural studies on conformational intermediates or drug discovery programmes where stabilisation of an inactive or partially-active intermediate receptor conformation is required. In contrast, GPCR-DIBMALP may provide a better platform for a screen in a discovery programme for a drug that fully activates the receptor. It would be important nevertheless, to confirm the conformational sub-state of individual GPCRs solubilised by SMA, SMI or DIBMA and not assume that all GPCRs behave exactly like rhodopsin. Furthermore, not all important GPCR conformational states will be accessible to investigation simply through choice of solubilising polymer alone.

## Conclusions

Nanodisc technology is becoming widely adopted as a strategy for solubilising membrane proteins due to the benefits provided over other solubilisation approaches. The direct solubilisation of proteins from membranes by SMALPs with the preservation of the native environment including annular lipids, has driven the generation of new SMA-like polymers resulting in SMILPs and DIBMALPs.

This study shows for the first time, that the dynamic range of conformational changes exhibited by a membrane protein encapsulated in a nanoscale lipid-particle is dictated by the chemical composition of the stabilising polymer employed to generate the nanoparticle. Using Rho, a member of the large GPCR family of membrane proteins that constitute the primary therapeutic target in drug discovery,^21^ we establish that SMA, SMI and DIBMA provide a ‘toolkit’ of solubilising polymers. Selection of the appropriate solubilising polymer provides a spectrum of useful attributes for studying membrane proteins, including generation of specific intermediate states and differences in thermostability of the encapsulated protein. This new insight into the expanded utility of the SMA-like polymers will have practical benefits for researchers studying membrane proteins using a wide range of approaches.

## Author contributions

MW, PJR, DRP, TRD and BK conceived the study. RLG, RTL, SAN, PS, PCE, CGT, PJR performed the methodology. The manuscript was written through contributions of all authors. All authors reviewed and edited the manuscript and have given approval to the final version.

## Conflicts of interest

There are no conflicts to declare.

## Supplementary Material

NR-013-D1NR02419A-s001
