# Supplementary material for: Differences in SMA-like polymer architecture dictate the conformational changes exhibited by the membrane protein rhodopsin encapsulated in lipid nano-particles
Source: Nanoscale. 2021 Aug 2;13(31):13519–28. doi: 10.1039/d1nr02419a (PMC8359648; doi:10.1039/d1nr02419a)
Supplement: NR-013-D1NR02419A-s001 [file NR-013-D1NR02419A-s001.pdf]

Electronic Supplementary Material (ESI) for Nanoscale.
This journal is © The Royal Society of Chemistry 2021

Electronic Supplementary Information

Differences In SMA-Like Polymer Architecture Dictate The Conformational Changes Exhibited By The Membrane Protein Rhodopsin Encapsulated In Lipid Nano-Particles.

Rachael L. Grime, [‡ab] *Richard T. Logan,* [‡a] *Stephanie A. Nestorow,*[a] *Pooja Sridhar,*[a] *Patricia C. Edwards,*[c] *Christopher G. Tate,*[c] *Bert Klumperman,*[d] *Tim R Dafforn,*[a] *David R. Poyner,*[e] *Philip J. Reeves,**[f] *and Mark Wheatley,**[gb]

[a] School of Biosciences, University of Birmingham, Birmingham, B15 2TT, UK

[b] Centre of Membrane Proteins and Receptors (COMPARE), University of Birmingham and University of Nottingham, Midlands, UK

[c]MRC Laboratory of Molecular Biology, Cambridge, CB2 0QH, UK

[d]Department of Chemistry and Polymer Science, Division of Polymer Science, Stellenbosch University, Private Bag X1, Matieland 7602, South Africa.

[e] Life and Health Sciences, Aston University, Birmingham B4 7ET, UK.

[f] School of Life Sciences, University of Essex, Wivenhoe Park, Essex CO4 3SQ, UK.

[g] Centre for Sport, Exercise and Life Sciences, Institute for Health & Wellbeing, Alison Gingell Building, Coventry University, Coventry, CV1 2DS, UK

‡These authors contributed equally to the work.

*Corresponding authors, e-mail: mark.wheatley@coventry.ac.uk

e-mail: preeves@essex.ac.uk

SUPPORTING EXPERIMENTAL

Expression and purification of mini-G_o

Expression and purification of mini-G_o (construct 12)[1] was performed as described previously for mini-G_s.[2] Briefly, mini-G_o was expressed from plasmid pET15b in *E. coli* strain BL21-CodonPlus(DE3)-RIL and purified from the *E. coli* lysate by Ni^{2+}-affinity chromatography. The His tag was subsequently removed by TEV cleavage and the mini-G_o further purified by collecting the flow-through from a Ni^{2+}-affinity column, concentration of the sample and then size exclusion chromatography. The purified mini-G_o was concentrated to 100 mg/mL and stored at -80˚C.

Effect of mini-G_o protein or Gt-peptide on Rho-LP rhodopsin preparations

Experiments with either mini-G_o or Gt-peptide (VLEDLKSCGLF) were performed in buffer containing 20 mM HEPES, 10 mM $MgCl_2$, 1 mM EGTA, pH 7.5. Rho-LP samples were incubated with mini-G_o at the molar ratio of 1:50 in the presence of apyrase (25 mU/mL; New England Biolabs) for 30 min. For the Gt-peptide, Rho-LP samples were incubated with 500 µM G(t)-peptide for 30 min. Samples were then analysed by UV-VIS absorption spectroscopy and the response to photobleaching characterised.

Preparation of DMPC-LPs

1,2-dimyristoyl-*sn*-glycero-3-phosphocholine (DMPC) was suspended in chloroform (1 mL of chloroform per 10 mg of lipid). Chloroform was evaporated from the lipid solution using an air-tap. The DMPC was resuspended in buffer (50 mM Tris, 150 mM NaCl, pH 7.5) using a sonicator water-bath for 1 min to yield a 10 mg/mL solution. The lipid solution was divided into 1 mL aliquots and subjected to 5 freeze-thaw cycles using a –80 °C freezer with a thaw step at 42 °C using a heat block. DMPC vesicles were solubilised by SMA, SMI or DIBMA as described for rod outer segment (ROS).

SUPPORTING DATA

Table S1. Solubilisation of rhodopsin from ROS by DIBMA. For each DIBMA concentration the extraction efficiency relative to the detergent DDM (1 %) is shown. Data are mean ± s.e.m., n = 3.

DIBMA concentration (%)	Extraction efficiency (%)
1.0	49 ± 18
2.5	74 ± 22
5.0	98 ± 5
10.0	107 ± 6

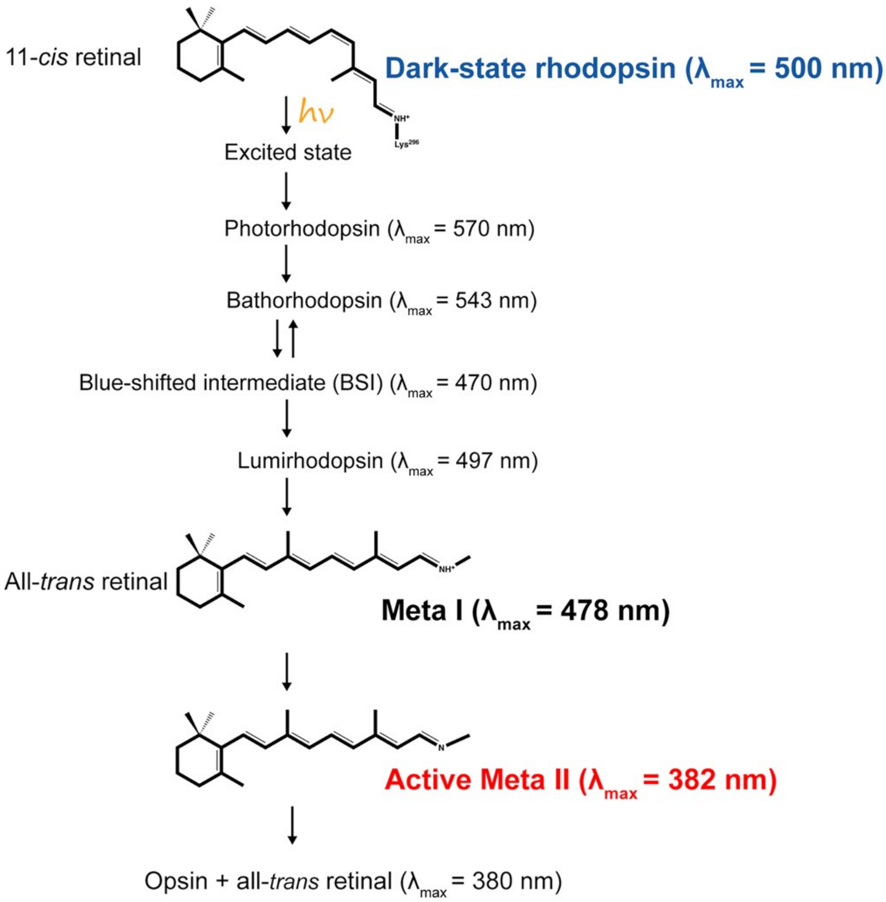

Fig. S1 Light-induced conformational changes of rhodopsin. Photointermediate states of rhodopsin between the inactive dark-state and active Meta II following activation by a photon of light, showing changes in the conformation of the chromophore between 11-*cis* retinal and all-*trans* retinal, and the characteristic absorption maximum (λ_{max}) of the structurally-defined intermediates.

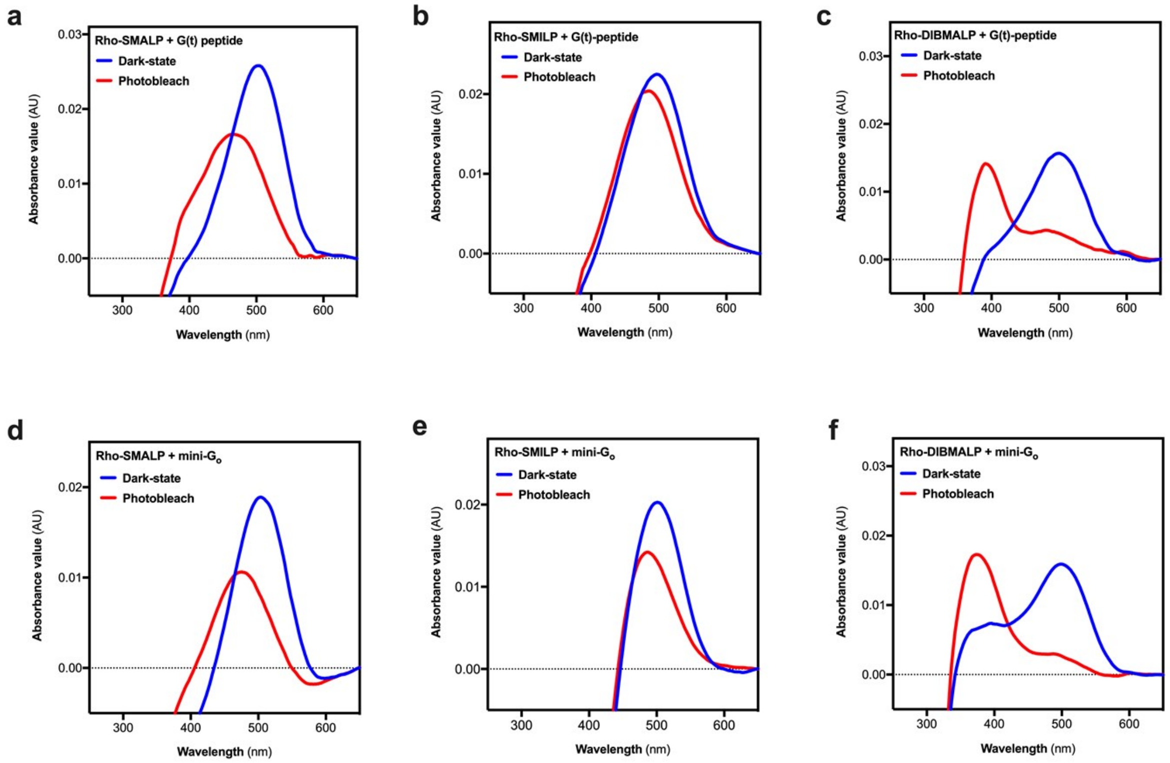

Fig. S2 Photoactivation of Rho-LP in the presence of G(t)-peptide or mini-G$_o$. Spectra of dark-state (blue), and photoactivated (red), rhodopsin encapsulated in a SMALP (a and d), SMILP (b and e) or DIBMALP (c and f) in the presence of G(t)-peptide (a – c) or mini-G$_o$ (d – f).

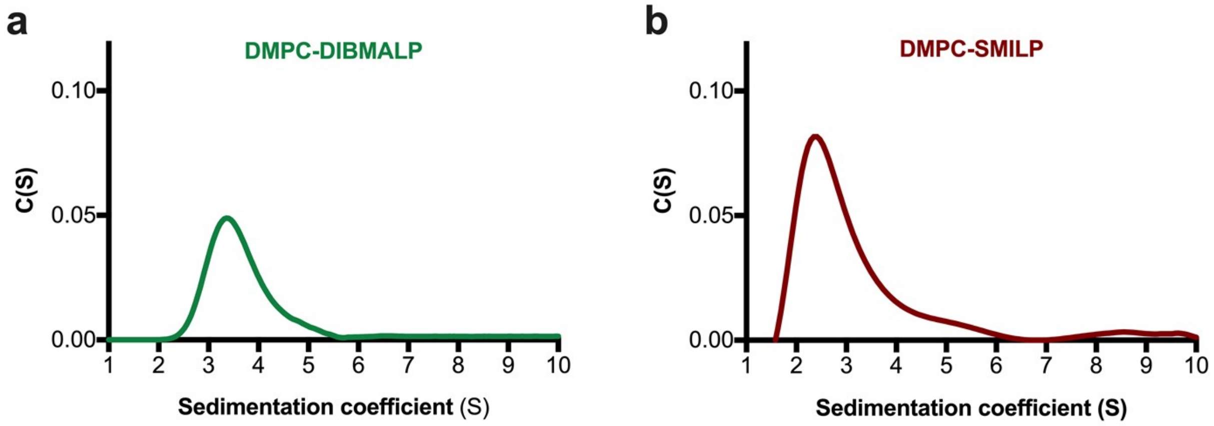

Fig. S3 Sedimentation velocity AUC analysis of DMPC-LPs. A representative AUC experiment is shown for (a) DMPC-DIBMALP and (b) DMPC-SMILP

SUPPORTING REFERENCES

1 R. Nehmé, B. Carpenter, A. Singhal, A. Strege, P. C. Edwards, C. F. White, H. Du, R. Grisshammer and C. G. Tate, *PLoS One,* 2017, **12**, e0175642.

2 B. Carpenter and C. G. Tate, *Bio Protoc.,* 2017**, 7**, e2235.
